# Case Report: Chronic Pulmonary Aspergillosis—An Unusual Long-Term Complication of Lung Cancer Treatment

**DOI:** 10.3389/fmed.2021.777457

**Published:** 2022-01-12

**Authors:** Katarzyna Guziejko, Katarzyna Klukowska, Urszula Budzińska, Robert Marek Mróz

**Affiliations:** ^1^2nd Department of Lung Diseases and Tuberculosis, Medical University of Bialystok, Białystok, Poland; ^2^Department of Medical Pathomorphology, Medical University of Bialystok, Białystok, Poland

**Keywords:** lung, cancer, aspergillosis, itraconazole, personalized treatment

## Abstract

**Background:** Chronic pulmonary aspergillosis (CPA) is a rare complication of radiochemotherapy for lung cancer. It may develop months or years after radical treatment. The diagnosis of CPA is challenging and complex. Not only fungal infection but also cancer relapse always have to be taken under consideration. Antifungal therapy is the base treatment, especially in the case when a surgical procedure is not possible. Standard treatment for at least 6 months is recommended but the optimal duration of the antifungal therapy is unknown. We present the clinical case of CPA, in which we had to perform multidirectional diagnostic tests to confirm the diagnosis and modified treatment due to the recurrence of the disease.

**Case Presentation:** We report a patient who developed CPA three and a half years after concurrent radiochemotherapy for locally advanced non-small-cell lung cancer. Non-specific symptoms were the cause of delayed diagnosis of fungal infection. Samples collected during bronchoscopy allowed to exclude the recurrence of lung cancer and establish the diagnosis of CPA. The patient was treated with itraconazole for 6 months. A few months later, controlled chest CT scans revealed the progression of CPA. Initially, retreatment with itraconazole was implemented. Due to the progression of fungal infection, voriconazole was used in the second line of treatment. Unfortunately, this therapy was complicated by the side effects and deterioration of the patient's condition. The reintroduction of itraconazole resulted in clinical and radiological improvement. Treatment is scheduled for at least 12 months.

**Conclusion:** Chronic pulmonary aspergillosis (CPA) was the cause of clinical deterioration and radiological progression in a patient after the radical treatment of lung cancer. In the described case, the diagnosis of CPA was delayed because of the suspicion of the recurrence of lung cancer. As the surgery was not possible, antifungal therapy with itraconazole was implemented and the proper dosage and duration led to significant clinical improvement.

## Introduction

Chronic pulmonary aspergillosis (CPA) is a rare disease (1). It is usually diagnosed in immunocompromised patients with other chronic respiratory disorders (2, 3). CPA risk factors include a history of pulmonary tuberculosis or non-tuberculous infections, lung cancer treated radically with surgery or radiochemotherapy, chronic lung disease, and emphysema (2–4). The diagnosis of CPA is challenging due to non-specific symptoms and it is based on clinical, radiological, and microbiological criteria, and the exclusion of other, more frequent causes of the reported symptoms (5, 6). Accurate diagnosis is crucial for initiating appropriate treatment. Antifungal treatment with itraconazole, voriconazole, and amphotericin B is recommended as a single or combination therapy in selected cases. The optimal duration of therapy is unknown (5). The duration of the treatment should be based on clinical and radiological improvement. In selected cases, surgical treatment is possible (5, 7, 8).

We present a history of a 54-year-old female patient, with adenocarcinoma of the right lung (cT3N2Mo CS IIIA), treated radically with chemoradiotherapy, who developed CPA as a long-term complication after oncological treatment. After the first six-month course of antifungal therapy, the disease recurred. The next course of treatment was complicated by side effects of the drug and affected the patient's quality of life.

## Case Description

**Figure 1:**
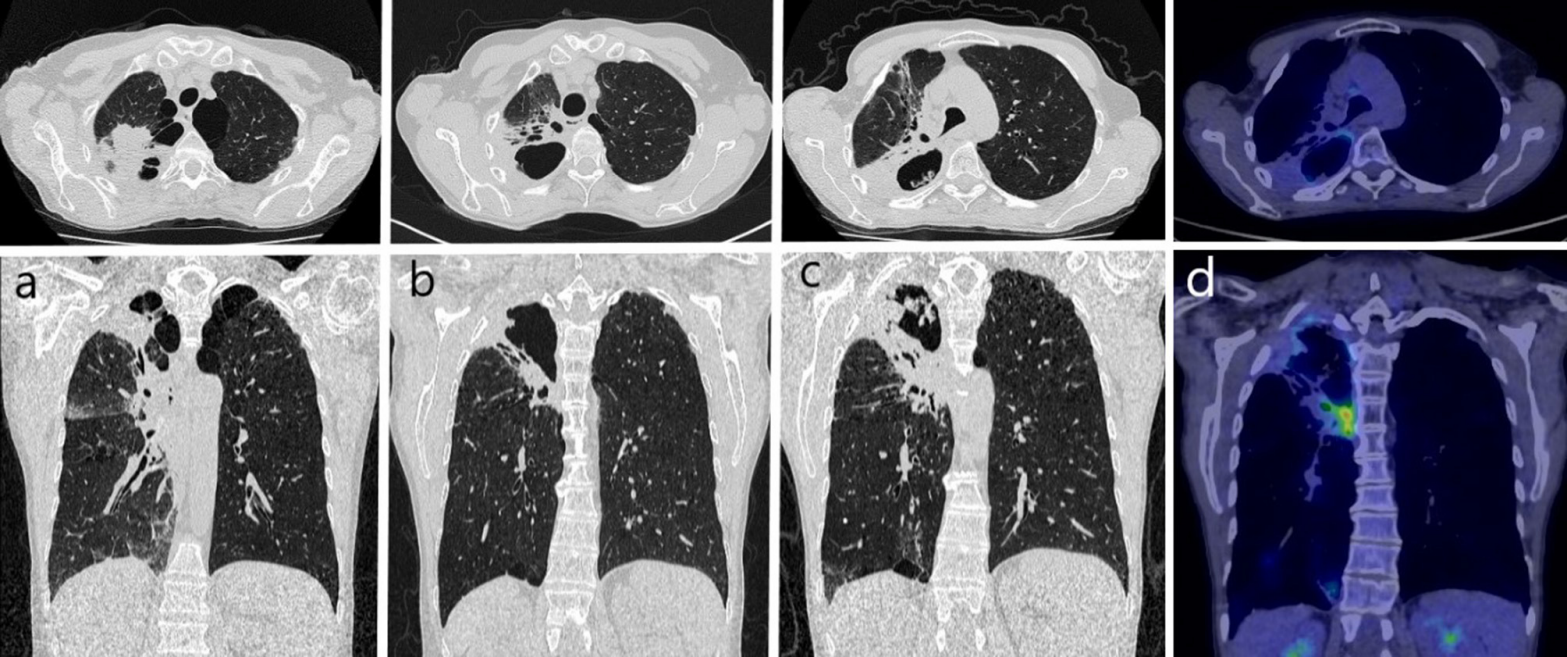
Chest CT in years 2016–2020: **(a)** 2016 - 3 months after chemoradiotherapy: solid tumor in the upper lobe of the right lung, emphysema in the apical parts of both lungs; **(b)** 2018 - cavity with the thick wall has formed at the site of the tumor in the right lung; **(c)** 2020 - progression of an irregular cavity in the upper part of the right lung with thick walls (concomitant mass on the posterior wall), surrounded by solid consolidation, dilated bronchus, and emphysema; **(d)** 2020 - positron emission tomography combined with computed tomography: unclear character of hypermetabolic masses in the right lung, more likely inflammatory, SUVmax = 2.5.

A 54-year-old female patient, active smoker (35 pack-years), with a history of pulmonary tuberculosis in adolescence, was admitted to the Department of Lung Diseases and Tuberculosis due to productive cough, weakness, low exercise tolerance, weight loss (about 7 kg). Symptoms exacerbated during last 6 months. She was treated empirically with oral antibiotics in standard doses (amoxicillin, amoxicillin-clavulanate, levofloxacin, and cefuroxime) without clinical improvement. Three and a half years earlier, the patient underwent radiochemotherapy for locally advanced non-small-cell lung cancer of the right upper lobe (adenocarcinoma, the clinical stage of the tumor was T3N2Mo CS IIIA /T—tumor, N—lymph node, M—metastasis/, Figure 1a). Platinum-based chemotherapy with etoposide (three cycles) was combined with radical radiotherapy of the right lung tumor area, hilum, and mediastinal lymph nodes (66 Gy in 33 fractions). This treatment was well-tolerated and no early complications were observed. Since then, the patient has been under strict oncological control. Follow-up CT images of the chest and abdomen were taken regularly every 6 months. A cavitary lesion developed at the site of the tumor of the right lung 2 years after the end of lung cancer treatment (Figure 1b).

Chest CT scans showed an irregular cavity in the upper part of the right lung with thick walls and accompanying mass of the posterior wall, surrounded by solid consolidation, dilated surrounding bronchus, and emphysema (Figure 1c). Imaging diagnostics was extended to PET-CT, which revealed the unclear nature of the hypermetabolic masses in the right lung, more likely inflammation (Figure 1d).

**Figure 2:**
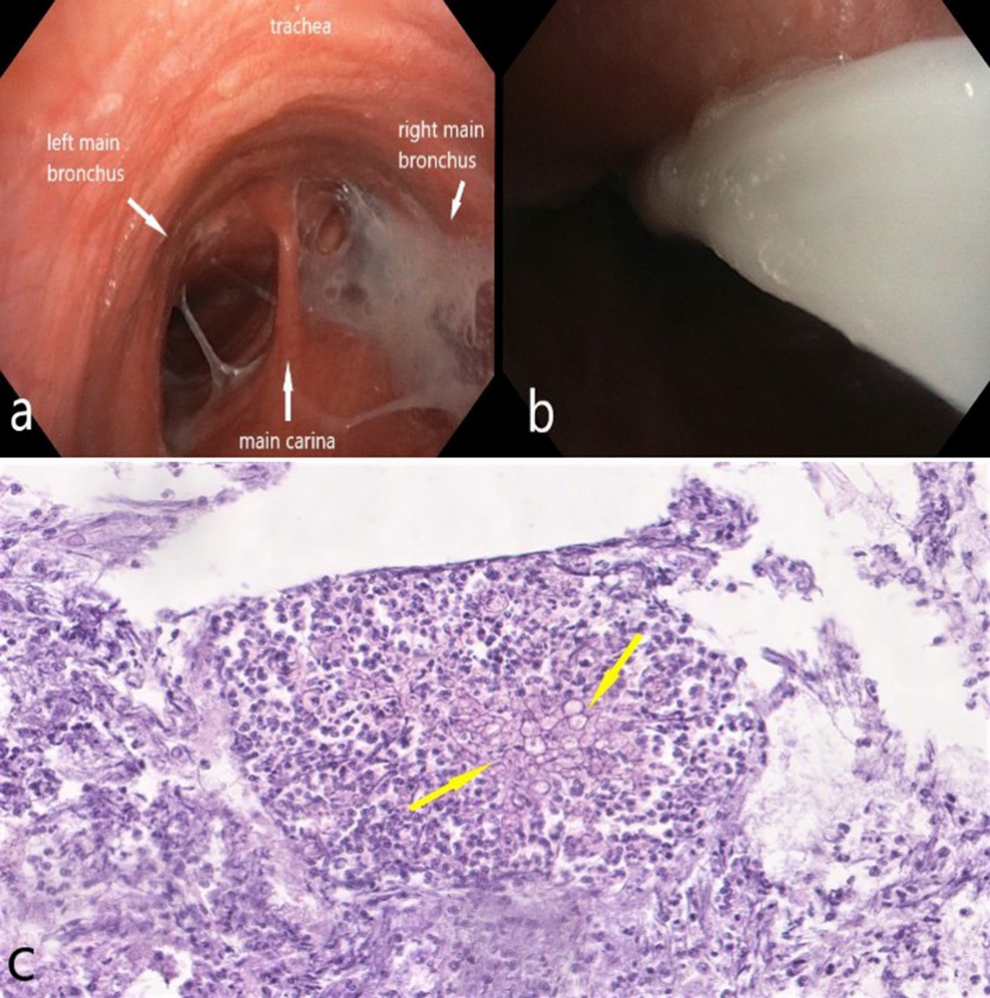
Bronchoscopy: **(a)** main trachea - purulent bronchitis, more severe in the right main bronchus; the bronchial mucosa is dull, red, with dilated vessels; **(b)** thick, purulent mucus in the right main bronchus; **(c)** fungal hyphae (yellow arrows) in the bronchial wash (original magnification × 100).

Physical examination revealed numerous wheezing and rales during lung auscultation. During bronchoscopy, purulent bronchitis was confirmed (Figures 2a,b). Laboratory tests showed increased concentration of C-reactive protein (CRP) (90 mg/dl, normal range <5 mg/dl). Lung function tests revealed a moderate impairment on pulmonary transfer coefficient for carbon monoxide (TLCO; 41% predicted value). No obstruction or restriction was observed.

Microbiological tests (smears, aerobic and anaerobic cultures) of the sputum and bronchial wash were negative for bacteria and fungi. Ziehl-Neelsen staining, cartridge-based nucleic acid amplification test (CBNAAT), and culture of sputum and bronchial wash did not show evidence of Mycobacterial infection. *Aspergillus fumigatus* precipitins were positive in serum. The test was performed using the qualitative Ouchterlony double immunodiffusion method in an agarose gel. The test was developed with its own research methodology and performed in the Department of Biological Health Hazards and Parasitology, Institute of Rural Health, Lublin, Poland. The result was assessed on the basis of the formed precipitation lines, validated with a negative control serum test. Ceftriaxone (2.0 g per day) and levofloxacin (500 mg twice a day) were administered empirically intravenously with a moderate clinical effect. Control bronchoscopy revealed a partial reduction of purulent mucus.

Due to the suspicion of lung cancer recurrence, a transthoracic, CT-guided fine-needle aspiration of the peripheral margin of the right lung cavity was performed. Only macrophages and inflammatory cells such as lymphocytes and neutrophils were observed in aspirated cytological samples. No microbiological tests were performed because of the small amount of the collected sample. Also, forceps biopsy of bronchial tissue collected from the second right bronchus during the bronchoscopy was negative for neoplastic cells. Moreover, no other clinical or radiographic signs of malignancy were observed.

The low value of transfer factor of the lung for carbon monoxide (TLCO) disqualified the patient from surgical resection of the right lung (pneumonectomy).

**Figure 3:**
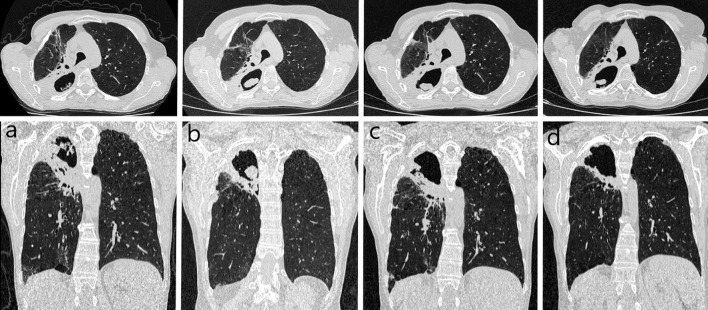
Controlled chest computed tomography during first-line treatment in 2020: **(a)** at the beginning of the therapy – irregular cavity size 102 x 59 mm in apical parts of the right lung, surrounded by solid consolidations widely adhere to the parietal pleura, connecting to the right hilum; **(b)** after 2 months – cavity with thick walls size 71 × 49 mm, concomitant mass on the posterior wall-size 29 x 10 mm; **(c)** after 4 months – cavity with thick walls size 69 × 47 mm, concomitant mass on the posterior wall-size 14 x 5 mm; **(d)** after 6 months – image stabilization.

**Figure 4:**
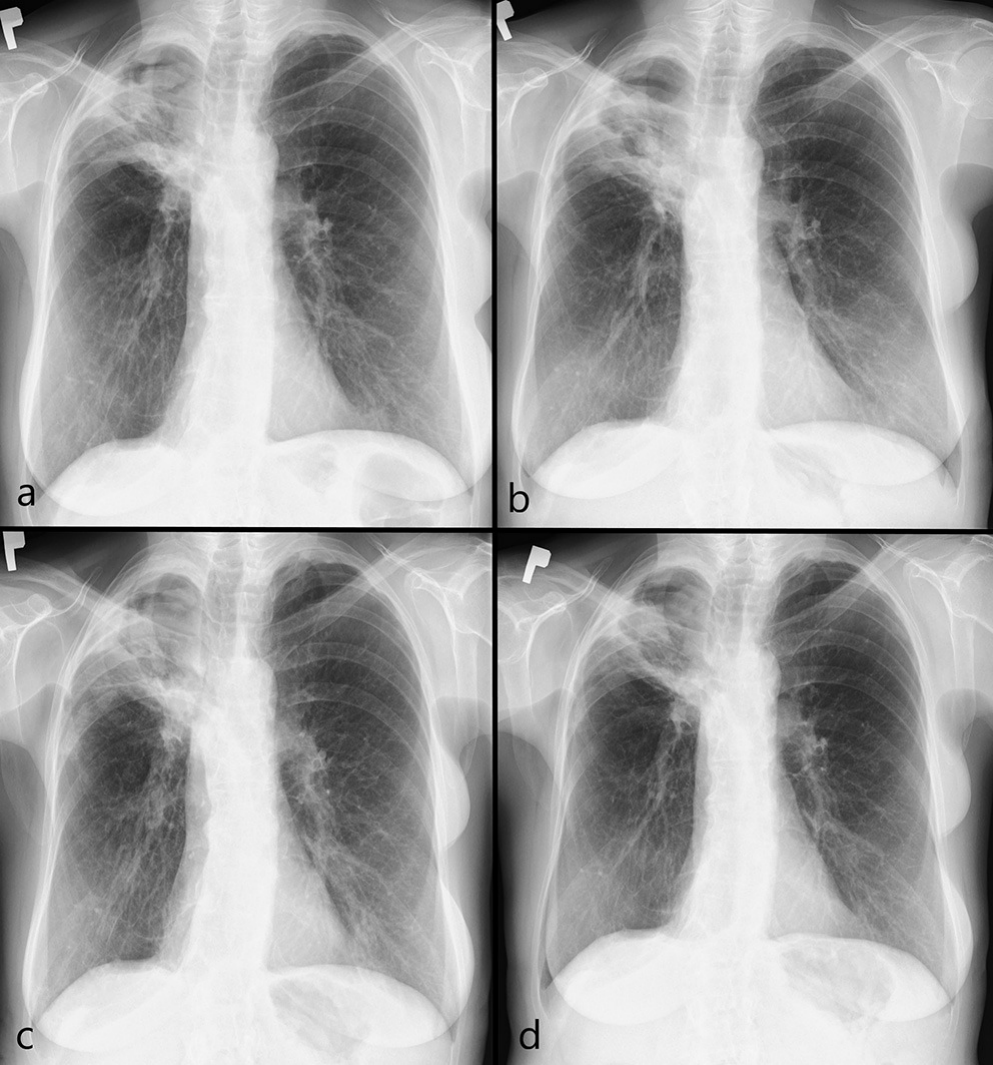
Controlled chest x-ray during first-line treatment in 2020: **(a)** at the beginning of the therapy – thick-walled cavity with internal consolidations in apical parts of the right lung; **(b)** after 2 months – cavity consolidation; **(c)** after 4 months – further cavity consolidation; **(d)** after 6 months – image stabilization.

The patient met diagnostic criteria for CPA and antifungal treatment with itraconazole was initiated (200 mg orally twice a day). The therapy was well-tolerated. Two months later, sputum culture was positive for *A. fumigatus*. A sabouraud dextrose agar (SDA) medium with chloramphenicol and gentamicin was used as a selective medium for the isolation of fungi. The species was determined on the basis of both macro- (colony morphology in SDA) and microscopic morphology (conidiophores, vesicles, metules, phialides, and conidia). Drug susceptibility was determined by the microdilution method (Thermo Scientific). The minimum inhibitory concentration (MIC) for itraconazole was 0.12 μg/ml. Every 8 weeks, the patient was re-evaluated clinically by physical examination and radiologically on the basis of chest CT (Figures 3a–d) and x-rays (Figures 4a–d). Follow-up bronchoscopy and microbiological tests of bronchial wash were also performed. Four months after initiation of itraconazole treatment, sputum culture was positive for itraconazole sensitive *A. flavus* (MIC = 0.25 μg/ml). Taking into account the well-graded clinical response, radiological improvement, and good tolerability, therapy was continued for up to 6 months.

**Figure 5:**
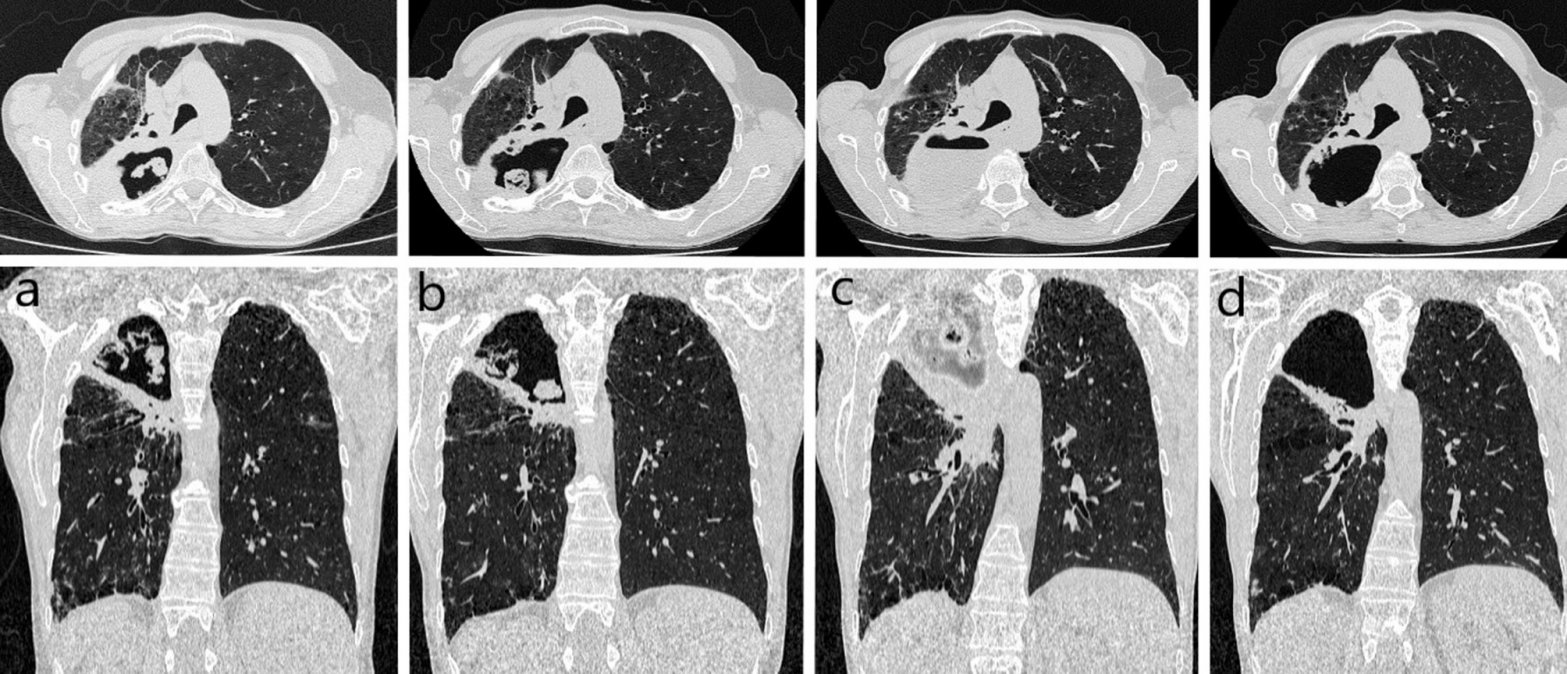
Controlled chest CT during second-line treatment in 2021: **(a)** at the beginning of the therapy – cavity with thick walls size 69 × 47 mm, concomitant mass on the posterior wall-size 22 × 9 mm, quantitative progression of the surrounding consolidations compared to the previous image; **(b)** after 3 months – cavity size 70 × 66 mm, the mass inside larger and more irregular (progression); **(c)** after 4 months – circumscribed opacity with an air-fluid level size 95 x 75 mm in the place of the previously described cavity (further progression, abscess); **(d)** after 5 months – cavity with thick wall much smaller, the mass inside size 28 × 20 mm, no fluid was visualized (regression).

**Figure 6:**
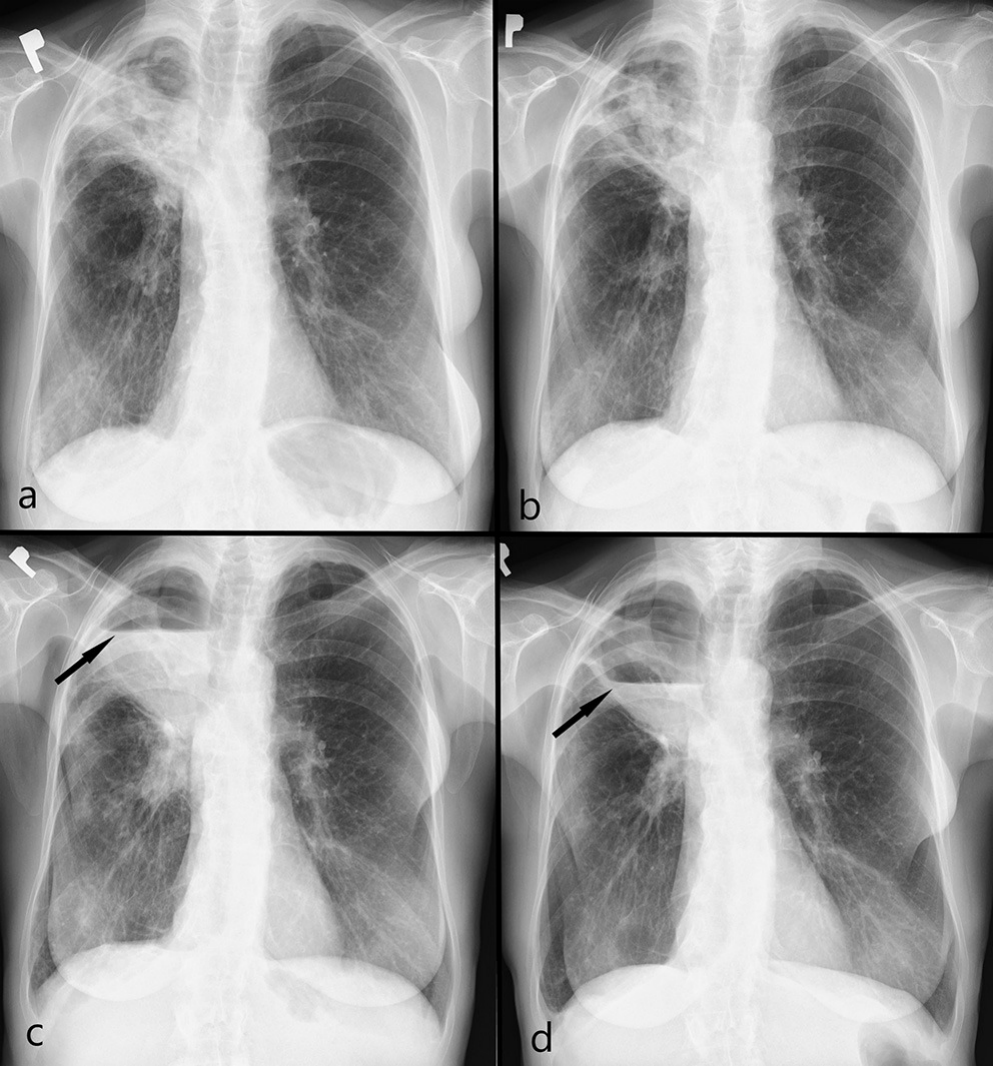
Controlled chest x-ray during second-line treatment in 2021: **(a)** at the beginning of the therapy - a thick-walled cavity in apical parts of the right lung, progression internal consolidations and surrounding opacities; **(b)** after 3 months – cavity consolidation, regression of surrounding opacities; **(c)** after 4 months – cystic lesion with an air-fluid level (arrow) (progression, abscess); **(d)** after 5 months – regression of an air-fluid level (arrow).

Six months later, the patient was re-admitted to the Department of Lung Diseases and Tuberculosis with symptoms of increasing weakness, productive cough, night sweats, chest pain on the right side, and weight loss (3 kg). Chest imaging confirmed CPA progression (Figures 5a, 6a). Microbiological tests for fungi and bacteria were negative. A bronchial biopsy ruled out cancer recurrence. Once again antifungal treatment with itraconazole (200 mg orally twice a day) was initiated. After 3 months, antifungal medication was switched to orally administered voriconazole (200 mg twice daily) due to radiographic progression (Figures 5b, 6b). The tolerance of the therapy was poor. Four weeks after starting the voriconazole treatment, the patient reported pain in the chest and right upper abdominal quadrant, further weight loss (a total of 7 kg), and temporary visual disturbances. On bronchoscopy, massive purulent bronchitis and concentric stenosis of the right upper lobe were detected. In the cytological examination of the bronchial wash, fungal hyphae were detected for the first time during the entire diagnostic process (Figure 2c). Chest CT images showed further progression. The cavity in the upper right lobe was larger, with a level of thickened, heterogeneous fluid and air in the upper part (Figures 5c, 6c). Physical examination and CT images of the abdominal cavity confirmed the hepatomegaly. The laboratory test revealed elevated transaminases (>5x upper limit of normal), high CRP (90 mg/dl), and anemia (red blood cells−3.4 x 10^6^/μl, hemoglobin−10.2g/dl).

The antifungal treatment was modified again. Itraconazole (200 mg orally twice a day) bridging therapy was used in anticipation of the unavailability of liposomal amphotericin B as part of emergency access to therapy.

**Figure 7:**
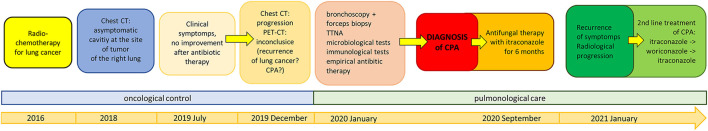
A timeline with all relevant data from this clinical case.

After 1 month of itraconazole treatment, clinical and radiological improvement was achieved. The patient confirmed her well-being, an increase in appetite and body weight (2 kg), and good tolerance of the antifungal therapy. Control imaging showed partial regression (Figures 5d, 6d). It was decided to continue treatment with itraconazole for at least 12 months under close clinical and radiological monitoring. The antifungal treatment is still ongoing. Follow-up visits are scheduled. A timeline with all relevant data from this clinical case is available in Figure 7.

## Discussion

Lung cancer is the most common cause of death in both men and women (9). The five-year survival rate for all stages of non-small lung cancer together is only around 20% which is rather disappointing (9). Follow-up after radical treatment should be based not only on monitoring the possibility of tumor recurrence but also on identifying possible late infectious complications in potentially immunosuppressed survivors.

CPA is a rare complication of lung cancer treatment. It is observed in patients who have undergone surgical resection or are treated radically with radiochemotherapy (4, 5, 10). The clinical course of CPA is chronic and progressive (5). Our patient developed CPA three and a half years after the completion of oncological treatment. CPA symptoms, such as coughing, weakness, night sweats, and weight loss are non-specific (5, 6). Therefore, they require multidirectional diagnostic methods in the field of imaging, endoscopy, microbiology, immunology, molecular biology, and pathology (5–7). Bronchoscopy, as a basic procedure in pulmonary patients, plays an important role in collecting samples for microbiological and histopathological examination. Cultures of bronchoalveolar lavage and bronchial washing fluid are more sensitive than spontaneously expectorated sputum cultures (5). For the diagnosis of CPA, culture and microscopy are essential. In patients with a high suspicion of CPA but with negative cultures samples, a supporting role is played by the detection of *Aspergillus* serum precipitin, IgG antibodies, and molecular tests such as PCR (5, 6, 11). In our patient, we had no direct evidence of *Aspergillus* infection but we confirm immunological response to *Aspergillus* species (7). The results of these tests brought us closer to the diagnosis of CPA and allowed to start the antifungal therapy.

The radiological features of CPA are the combination of the findings resulting from the underlying lung disease and changes secondary to the ongoing infection (12). CPA most often occurs as one or more pulmonary cavities possibly containing one or more aspergillomas, as well as irregular intraluminal material. Accompanying pleural thickening, surrounding consolidations, and adjacent bronchiectasis have also been reported (5, 7). To confirm the diagnosis, radiological progression confirmed after at least 3 months of follow-up observation is required (5). Our patient's CT and PET-CT images were ambiguous and progressive. They suggested a recurrence of lung cancer or CPA.

Azoles are the first-line drug for CPA. In the second line of treatment, liposomal amphotericin B or echinocandins are used (5, 7). The optimal duration of the antifungal therapy is unknown. Standard treatment for at least 6 months is recommended (7, 8). The implementation of the therapy results in a slow but gradual clinical recovery and radiological improvement (5). Bongomin et al. suggest that extended therapy may be required to achieve clinical improvement (8). They demonstrated among a cohort of 206 patients with CPA that azoles are modestly effective, especially if given for 12 months. After 6 months of treatment with itraconazole, the described patient had a recurrence of the infection. Therefore, the duration of the second-line treatment is planned for at least 12 months. The use of alternative drugs such as liposomal amphotericin B was limited due to a lack of availability at the time.

Antifungal treatment is often associated with side effects. Common side effects during antifungal treatment are hepatotoxicity, cytopenia, photosensitivity, gastrointestinal symptoms, and allergic reactions (1). Therefore, careful monitoring is crucial for them. The frequency of monitoring should be based on the patient's age, concomitant medications, comorbidities, drug toxicities, and resources. Immediate identification and management of side effects reduce the risk of treatment and possibly improve changes at the end of treatment (11, 13). Despite strict monitoring in the described case, treatment with voriconazole had to be discontinued due to hepatotoxicity.

For symptomatic patients unresponsive to medical therapy, surgical resection of simple aspergilloma is strongly recommended (6). The type of surgery should be based on clinical indications, the extent of the disease, and the results of pulmonary function tests (5). If the estimated postoperative TLCO is <40% of predicted, the risk of morbidity and mortality is quite significant (14). In our case, surgical treatment was not possible due to the high risk of postoperative respiratory failure.

## Conclusion

Chronic pulmonary aspergillosis (CPA) was the cause of clinical deterioration and radiological progression in a patient after radical treatment of lung cancer. In the described case, the diagnosis of CPA was delayed because of the suspicion of recurrence of lung cancer. As the surgery was not possible, antifungal therapy with itraconazole was implemented and the proper dosage and duration led to significant clinical improvement.

## Data Availability Statement

The original contributions presented in the study are included in the article/supplementary material, further inquiries can be directed to the corresponding author/s.

## Ethics Statement

Written informed consent for the publication of identifying images or other personal or clinical details of participant that compromise anonymity was obtained.

## Author Contributions

KG and RM: analyzed and prepared the data and wrote the manuscript. UB: performed the pathomorphological study. KK: was the leading doctor. All authors read and approved the final version of the manuscript.

## Conflict of Interest

The authors declare that the research was conducted in the absence of any commercial or financial relationships that could be construed as a potential conflict of interest.

## Publisher's Note

All claims expressed in this article are solely those of the authors and do not necessarily represent those of their affiliated organizations, or those of the publisher, the editors and the reviewers. Any product that may be evaluated in this article, or claim that may be made by its manufacturer, is not guaranteed or endorsed by the publisher.

## Abbreviations

CBNAAT: cartridge-based nucleic acid amplification test
CPA: chronic pulmonary aspergillosis
CRP: C-reactive protein
CT: computed tomography
MIC: minimum inhibitory concentration
PCR: polymerase chain reaction
PET-CT: positron emission tomography combined with computed tomography
SDA: Sabouraud dextrose agar
TLCO: transfer coefficient for carbon monoxide.
